# UPLC Quantitative Analysis of Multi-Components by Single Marker and Quality Evaluation of *Polygala tenuifolia* Wild. Extracts

**DOI:** 10.3390/molecules22122276

**Published:** 2017-12-20

**Authors:** Rui Xu, Fuying Mao, Yunsheng Zhao, Wenping Wang, Lingling Fan, Xiaojuan Gao, Jianjun Zhao, Hongling Tian

**Affiliations:** 1 School of Pharmacy, Ningxia Medical University, Yinchuan 750004, Ningxia, China; woshixurui925@163.com (R.X.); mfyzys@126.com (F.M.); wpwang2015@126.com (W.W); fll911215@163.com (L.F.); gaoxiaojuan0705@126.com (X.G.); zhaojianjun777@126.com (J.Z.); 2 Ningxia Research Center of Modern Hui Medicine Engineering and Technology, Yinchuan 750004, Ningxia, China; 3 Key Laboratory of Hui Ethnic Medicine Modernization, Ministry of Education, Yinchuan 750004, Ningxia, China; 4 Institute of Industrial Crop Research, Shanxi Academy of Agricultural Sciences, Fenyang 032200, Shanxi, China; thl2003@163.com

**Keywords:** *Polygala tenuifolia* Wild., multi-components by single marker, ultra-performance liquid chromatography, quality evaluation

## Abstract

The quality control of *Polygala tenuifolia* Wild. is a major challenge in its clinical application. In this paper, a new strategy for the quality evaluation of *P. tenuifolia* extracts was verified through reverse-phase ultra-performance liquid chromatography (UPLC). The quantitative analysis of multi-components by a single marker (QAMS) was conducted with 3,6′-disinapoyl sucrose as an internal reference substance. Eight components (i.e., sibiricose A5, sibiricose A6, glomeratose A, tenuifoliside A, tenuifoliside B, tenuifoliside C, sibiricaxanthone B, and polygalaxanthone III) were determined based on the relative correction factors. The concentrations of these components were also determined by applying a conventional external standard method. The cosine value confirmed the consistency of the two methods (cosine ratio value >0.999920). Hierarchical cluster analysis, radar plots, and discriminant analysis were performed to classify 23 batches of *P. tenuifolia* extracts from Shanxi, Hebei, and Shaanxi in China. Results revealed that QAMS combined with radar plots and multivariate data analysis could accurately measure and clearly distinguish the different quality samples of *P. tenuifolia*. Hence, QAMS is a feasible and promising method for the quality control of *P. tenuifolia*.

## 1. Introduction

Traditional Chinese medicine (TCM) has gained increasing attention worldwide; in this regard, ensuring the curative effects of TCM has become an important issue [1,2]. The quality control of TCM has depended on only one single indicator, which cannot reflect its pleiotropy and integrity. At present, the simultaneous determination of multi-components (SDMC) is used to characterize herbal or botanical products. Chemical fingerprint analysis is also used to evaluate the quality of herbal drugs, identify their authenticity, and differentiate their origins [3]. As an important part of fingerprint analysis, reverse-phase ultra-performance liquid chromatography (UPLC) exhibits significant higher peak resolution, sensitivity, and speed of analysis than high-performance liquid chromatography (HPLC) [4,5]. UPLC utilizes high linear velocities, with columns packed with particles that are less than 2 μm, and operates at high pressure [6,7]. The UPLC system has been used in various pharmaceutical products [8]. However, few studies have used UPLC to analyze *Polygala tenuifolia* extracts and its active components. SDMC includes two main methods based on conventional chemical fingerprint analysis. First, the normal external standard method (ESM) uses multiple reference standards to analyze multiple components. The use of this model is a more scientific and reasonable approach than utilizing a single component as an indicator. However, this strategy utilizes expensive and scarce high-purity (98%) standard substances, thereby restricting its application [9,10]. The high testing fees of a multiple index control limit the scientific research and supervised application of the ESM. To address these limitations, scholars have developed a second approach, namely, quantitative analysis of multi-components by a single marker (QAMS), which requires a single reference standard, in order to simultaneously determine the concentrations of multi-components. The QAMS model was established to study the quality control of TCM, and has been widely applied [11,12,13]. This method not only significantly reduces the cost and detection time of the experiment, it also improves its practicability.

Polygalae Radix, which is called Yuanzhi in Chinese, was originally recorded in *Shen Nong’s Herbal Classic* as one of the oldest and still most commonly used TCMs, and is officially listed in the Chinese pharmacopoeia [14,15]. Polygalae Radix is the dry root of *P. tenuifolia* Wild. or *P. sibiric* L., both of which belong to the Polygalaceae family. *P. tenuifolia* is the most dominant origin plant of Polygalae Radix [16], and is an ingredient in numerous medicines and prescriptions in East Asia [17]. Based on laboratory studies and clinical practice, *P. tenuifolia* possesses various biological and therapeutic actions and functions as a mucolytic, tonic, sedative, antipsychotic, and expectorant [14,18,19]. Phytochemical studies of the dried roots of *P. tenuifolia* revealed that saponins, xanthones, and oligosaccharides are the major and bioactive secondary metabolites [20,21,22]. Saponins are considered active ingredients of expectorants, antitussives, and short-term memory enhancers [19,23]. Xanthones exhibit anti-tumor [24], anti-inflammation [25], anti-thrombotic [26], and antimicrobial activities [27]. The total oligosaccharide esters of *P. tenuifolia* are used as an antidepressant [28,29]. In the current Chinese pharmacopoeia, 3,6′-disinapoyl sucrose (DISS), polygalaxanthone III, and tenuifolin are listed as the indicators for assessing the quality of *P. tenuifolia*. Although these three indicators are used to evaluate the quality of *P. tenuifolia*, other important ingredients have been ignored, resulting in insufficient information on the integral chemical composition of *P. tenuifolia*. Therefore, a simple, comprehensive, and effective method must be developed for evaluating the quality of *P. tenuifolia*.

The clinical efficacy of *P. tenuifolia* depends on the joint actions of multiple effective constituents, and quality control is characteristic of integrity and diversity, so one or three single indicators cannot reflect its quality well. SDMC is a more scientific and reasonable approach than determining a single indicator by the ESM. However, this strategy needs many expensive standard substances and a lot of time, thereby limiting its application. QAMS is able to simultaneously determine the concentrations of multi-components by UPLC, and only requires a single reference standard, which significantly reduces the cost and detection time of the experiment. Therefore, the present study: (1) established a simple, economic, and accessible UPLC–QAMS method based on nine components for the quality evaluation of *P. tenuifolia*.; (2) assessed the applicability and feasibility of the methodology by the angle cosine value between QAMS and the ESM; and (3) evaluated the quality of 23 batches of *P. tenuifolia* extracts by UPLC–QAMS combined with hierarchical cluster analysis, radar plot analysis, and discriminant analysis.

## 2. Results and Discussion

### 2.1. The Establishment of Analytical Protocol

#### 2.1.1. Blank Control

The blank tests were conducted with methanol or 70 *v*/*v* methanol to eliminate the systematic or operating error caused by the laboratory environment, glassware, materials, or operators. The nine standard substances and other unknown constitutions were all not detected. So, the experiment error did not affect the subsequent qualitative and quantitative analysis.

#### 2.1.2. Representative UPLC Chromatograms

**Figure 1:**
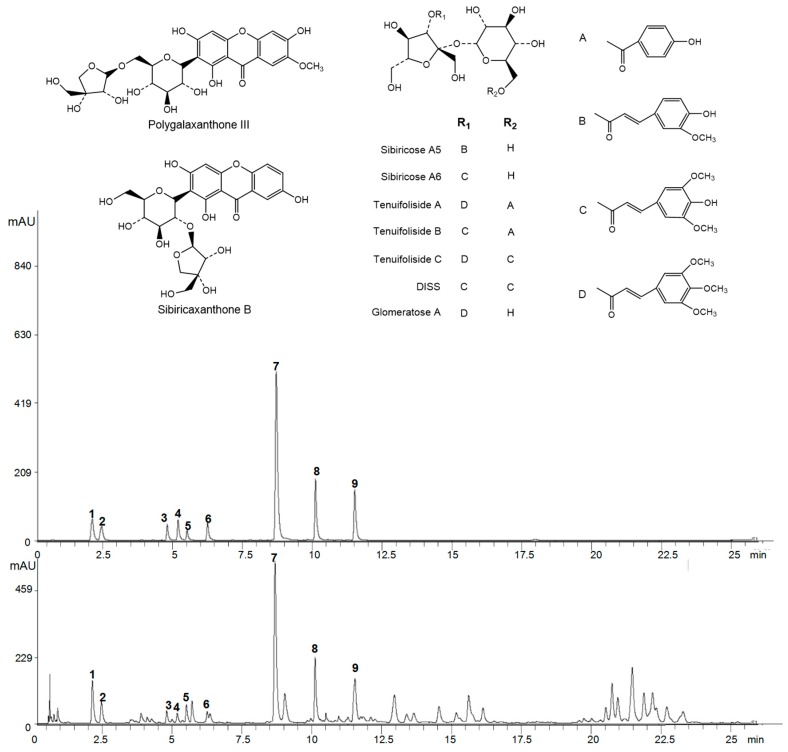
Chromatograms of a sample of *P. tenuifolia* and nine mixed reference standards. Note: 1: sibiricose A5; 2: sibiricose A6; 3: sibiricaxanthone B; 4: glomeratose A; 5: polygalaxanthone III; 6: tenuifoliside B; 7: 3,6′-disinapoyl sucrose (DISS); 8: tenuifoliside A; 9: tenuifoliside C.

A detection wavelength of 320 nm was selected for the quantitative analysis of the nine components on the basis of the stable baseline, non-peak interference, and maximum absorption. Gradient elution was developed for the effective separation of *P. tenuifolia*. Under these chromatographic conditions, a good separation was achieved within 26 min for the *P. tenuifolia* extracts and the standard mixture. The chromatograms are shown in Figure 1. According to the relative retention times of the reference chromatograms, peaks 1–9 were identified as sibiricose A5, sibiricose A6, sibiricaxanthone B, glomeratose A, polygalaxanthone III, tenuifoliside B, DISS, tenuifoliside A, and tenuifoliside C, respectively.

#### 2.1.3. The Building of Calibration Curves

The calibration curves of the nine standard substances were plotted with a series of injection volumes of standard solutions. As shown in Table 1, the high correlation coefficient values (R^2^ > 0.9990) displayed good linearity at a wide range of concentrations. The limit of quantification (LOQ) and limit of detection (LOD) values were determined by using signal-to-noise ratios of 3:1 and 10:1, respectively. The method detection limit (MDL) was measured by analyzing seven negative samples spiked with nine standard substances at a concentration level that provided an approximated signal-to-noise ratio between 2.5 and 5, which was calculated using the equation: MDL = (SD) × t (*n* − 1,1 − α = 0.99), where SD is the standard deviation of the replicate analyses, t (*n* − 1,1 − α = 0.99) is Student’s t value for a 99% confidence level and a SD estimate with *n* − 1 degrees of freedom (t = 3.14 for seven replicates), and *n* is the number of replicates. The method quantitation limit (MQL) was calculated by multiplying the SD of the results that were used to determine MDL by 10 [30]. The linearity, LODs, LOQs, MDL, and MQL for each investigated compound showed a high sensitivity under the chromatographic conditions, implying that the obtained calibration curves could be applied to QAMS analysis. 

#### 2.1.4. Method Validation

Method precision was evaluated based on intra-day and inter-day variability. Intra-day variability was conducted by injecting the *P. tenuifolia* sample (**S1**) solution six consecutive times in the same day. Inter-day variability was realized by using the same solution and injection times for two successive days. Intermediate precision was determined by analyzing the *P. tenuifolia* sample (**S1**) solution at random events including days, analysts, and equipment (relative peak area of DISS). The obtained relative standard deviation (RSD) values are summarized in Table 2. As shown, the results were precise for intra-day variability (with RSD values between 0.19% and 0.83%), inter-day variability (with RSD values between 0.40% and 1.07%), and intermediate precision (with RSD values between 0.19% and 0.83%). Stability was determined by analyzing the *P. tenuifolia* sample (**S1**) solution at 0, 2, 4, 8, 10, 12, and 24 h at room temperature: (26 ± 2 °C). The sample solutions were stable within 24 h (RSD ≤ 2.86%). Furthermore, the repeatability test was performed through six injections of independently prepared sample **S1** solutions, and the result revealed that the RSD was between 1.34 and 3.27%. The recovery was assessed by adding a known amount of individual standards into a certain amount (0.50 g) of *P. tenuifolia* sample (**S1**), and the variations of the concentrations were expressed as RSD values of the peak area (PA). The mixture was extracted and analyzed by using the aforementioned method. The average recoveries were in the range of 95.48–101.90%. Six replicates were performed for each analysis. All of the results suggested that these methods were effective and reliable. 

#### 2.1.5. Calculation of the Relative Calibration Factors

**Figure 2:**
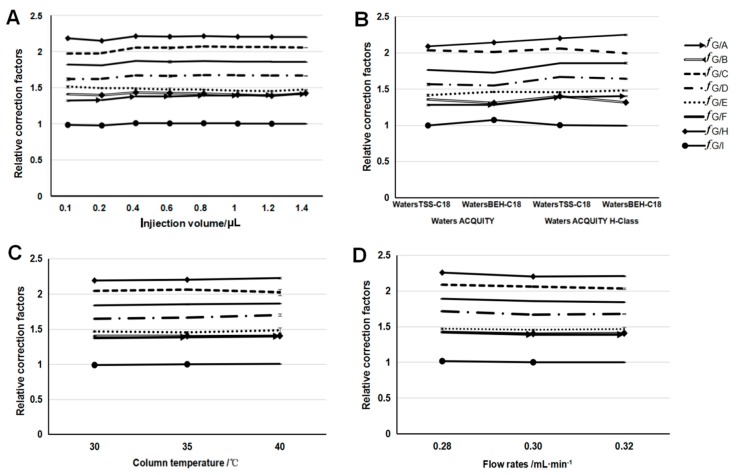
Results of the relative calibration factors (RCFs) and robustness test (mean ± SD, *n* = 3). (**A**) RCFs of different injection volumes; (**B**) RCFs of different instruments and different columns; (**C**) RCFs of different column temperatures; (**D**) RCFs of different flow rates in the mobile phase. Note: *f*G/A: sibiricose A5; *f*G/B: sibiricose A6; *f*G/C: sibiricaxanthone B; *f*G/D: glomeratose A; *f*G/E: polygalaxanthone III; *f*G/F: tenuifoliside B; *f*G/H: tenuifoliside A; *f*G/I: tenuifoliside C.

The same mixed reference substance solutions of the nine components were tested with a series of injection volumes to identify the relative calibration factors (RCFs) (Figure 2A). DISS was selected as the internal reference substance to calculate the RCFs by using Equation (5). Figure 2A shows that the RCFs of different injection volumes (0.1–1.4 µL) from one component were similar for the specific reference substance. Then, the mean RCFs were calculated as the final RCFs of sibiricose A5 (1.371), sibiricose A6 (1.408), sibiricaxanthone B (2.033), glomeratose A (1.652), polygalaxanthone III (1.475), tenuifoliside B (1.845), tenuifoliside A (2.191), and tenuifoliside C (0.995) (Supplementary Table S1).

#### 2.1.6. Robustness of the Relative Calibration Factors

To assess the robustness of the RCFs, the influences of the different instruments (Waters ACQUITY and Waters ACQUITY H-Class), different columns (TSS-C18 and BEH-C18), different column temperatures (30 °C, 35 °C, and 40 °C), and different flow rates of the mobile phase (0.28, 0.30, and 0.32 mL·min^−1^) were investigated (Figure 2B–D). The line chart results show that these influencing parameters did not exert any considerable influence on the RCFs, all of which had RSDs lower than 5% (Supplementary Tables S2 and S3). Therefore, the QAMS method is suitable for quantifying multi-components with good adaptability and durability when standard substances are unavailable.

#### 2.1.7. Similarity Evaluation of the QAMS and ESM Methods

To evaluate and verify the feasibility of QAMS for determining the multi-components in *P. tenuifolia*, 23 representative samples from different habitats were used to evaluate the similarity. The concentrations of the nine components were determined through the ESM and the QAMS method in 23 batches. The angle cosine value (*C_ir_*), which ranges from 0 to 1, is commonly used to evaluate the similarity of TCM fingerprints. A larger *C_ir_* value indicates a higher similarity of the target samples. When the *C_ir_* is equal to 1, the targets are identical [31]. The results (Table 3) revealed that *C_ir_* exceeded 0.999920, suggesting that no significant differences existed between the ESM and the QAMS method. Thus, the proposed QAMS method could well detect the concentration of each component and feasibly quantify the compounds of *P. tenuifolia.*

### 2.2. Quality Evaluation of P. tenuifolia Based on Nine Components

#### 2.2.1. Concentrations of the Nine Components in the *P. tenuifolia* Samples

The concentrations of the nine components in 23 batches of *P. tenuifolia* obtained by the QAMS method are listed in Table 3. Among the nine components, DISS (5.523 mg/g) and tenuifoliside A (3.524 mg/g) displayed higher mean concentrations in *P. tenuifolia*, whereas sibiricaxanthone B (0.867 mg/g), glomeratose A (0.786 mg/g), polygalaxanthone III (0.577 mg/g), and tenuifoliside B (0.471 mg/g) had lower mean concentrations. For all 23 batches, the analyte of the highest concentration was DISS (7.15 ± 0.29 mg/g) in producers S2, and the analyte of the lowest concentration was tenuifoliside B (0.31 ± 0.02 mg/g) in S18. The maximum and minimum total concentrations of the nine components were 21.291 and 12.053 mg/g, respectively. The data in Table 3 presented differences among various sample pairs. To show the clear classification of the *P. tenuifolia* samples, the QAMS method with chemometrics analysis was performed in the subsequent analyses.

#### 2.2.2. Hierarchical Clustering Analysis (HCA)

**Figure 3:**
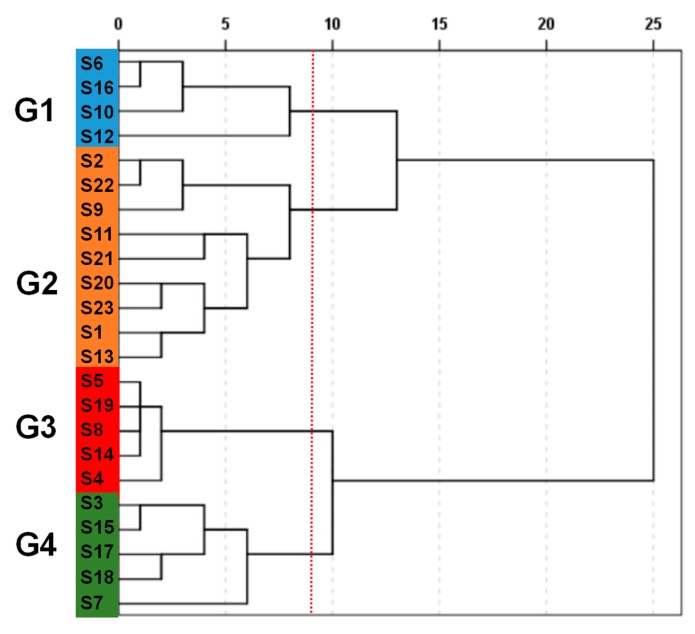
Dendrogram of nine chemical compositions for *P. tenuifolia* of different origins. G1: group 1; G2: group 2; G3: group 3; G4: group 4.

To quantitatively and objectively analyze *P. tenuifolia*, hierarchical clustering analysis (HCA) was performed according to the concentrations of the nine components, and a dendrogram was obtained. As shown in Figure 3, the 23 samples from different origins could be divided into four distinct groups (G1–G4) at the rescaled distance of nine. This process was subjective and non-quantitative. S6, S10, S12, and S16 were grouped under G1. S1, S2, S9, S11, S13, S20, S21, S22, and S23 were assigned to G2. S4, S5, S8, S14, and S19 were placed under G3. S3, S7, S15, S17, and S18 were classified under G4. The results showed that HCA can classify the similarity of *P. tenuifolia* on the basis of the concentrations of the nine components. However, HCA failed to clearly indicate which group had a high quality. Therefore, radar plot analysis was used in the following quality analysis.

#### 2.2.3. Radar Plot Analysis 

**Figure 4:**
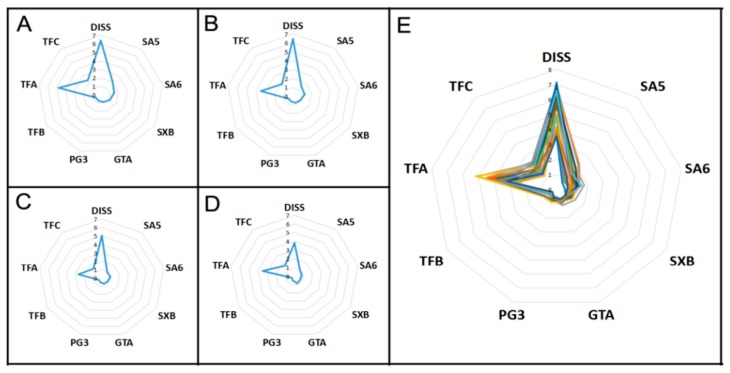
Radar plots showing the difference of geographical origins in terms of nine components in various *P. tenuifolia* samples; (**A**) G1; (**B**) G2; (**C**) G3; and (**D**) G4. **E**: the distribution of the chemical composition patterns of 23 *P. tenuifolia* samples. Note: DISS: 3,6′-disinapoyl sucrose; SA5: sibiricose A5; SA6: sibiricose A6; SXB: sibiricaxanthone B; GTA: glomeratose A; PG3: polygalaxanthone III; TFB: tenuifoliside B; TFA: tenuifoliside A; TFC: tenuifoliside C.

A radar plot was used to assess the quality of *P. tenuifolia* samples because of its simple, rapid, and routine discrimination. For ease of comparison, the radar plot was employed to preliminarily classify *P. tenuifolia* samples on the basis of the concentrations of the nine components. Radar plot analyses were conducted on 23 *P. tenuifolia* samples. Figure 4A–D show the means of the nine components in G1–G4 in the HCA, respectively. As shown, the distributions of the nine components of the *P. tenuifolia* samples from various groups exhibited different characteristic patterns. The samples from G1 and G2 had a distinctly higher concentration of the nine components and were easily discriminated compared with the *P. tenuifolia* samples from the other two groups. Therefore, radar plot analysis could distinguish the quality of the different *P. tenuifolia* samples. As shown in Figure 4E, the distribution of the chemical composition patterns displayed similar characteristics. This finding indicated that the *P. tenuifolia* samples from the various localities had similar features because of species heredity. However, visual measurement was the only result received by radar plot analysis, and the lack of clear indicators describing the exact distinctions reduced the dependability of the results. Therefore, a model should be built to distinguish the quality of unknown samples on the basis of the concentrations of the nine components. Therefore, discrimination analysis (DA) was utilized in the following studies to achieve actual discrimination.

#### 2.2.4. Discrimination analysis (DA) 

The nine components were selected as variables for discriminant analysis to classify the *P. tenuifolia* samples. Then, three discriminant functions (F1–F3) and four Fisher’s linear classification functions that correlated with the variables were obtained. The classification functions were as follows:

G1 = 271.95X1 − 508.73X2 + 207.15X3 + 427.79X4 + 182.49X5 + 642.53X6 + 272.69X7 + 40.79X8 + 36.86X9 − 1488.32,
(1)

G2 = 221.37X1 − 451.23X2 + 158.84X3 + 355.53X4 + 204.25X5 + 560.04X6 + 254.57X7 + 40.42X8 + 25.18X9 − 1196.75,
(2)

G3 = 170.74X1 − 353.18X2 + 135.05X3 + 285.06X4 + 168.98X5 + 436.00X6 + 200.12X7 + 29.62X8 + 21.77X9 − 748.5,
(3)

G4 = 172.30X1 − 327.78X2 + 91.30X3 + 306.93X4 + 143.41X5 + 382.30X6 + 173.63X7 + 33.04X8 + 19.62X9 − 614.96,
(4)

According to the classification standard, the nine variables that corresponded to the concentrations of the nine components generated four Fisher’s linear classification functions. Each sample had four functional values, and was assigned to the corresponding group with the highest value. Sibiricose A5, sibiricose A6, sibiricaxanthone B, polygalaxanthone III, glomeratose A, tenuifoliside B, DISS, tenuifoliside A, and tenuifoliside C were represented by X1–X9, respectively. For unknown samples, the nine variables were brought into the equations (Equations (1)–(4)), and the unknown samples were grouped according to the obtained classification standard values.

**Figure 5:**
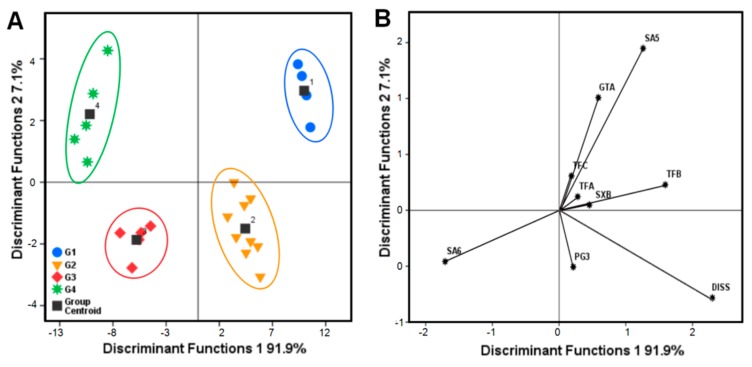
Quality discrimination analysis (DA) for *P. tenuifolia* samples from three different provinces. **A**: Scatter diagram of *P. tenuifolia* samples from four different groups (G1, G2, G3, and G4); **B**: Correlation chart between the variables and the discriminant functions for all of the *P. tenuifolia* samples. Note: DISS: 3,6′-disinapoyl sucrose; SA5: sibiricose A5; SA6: sibiricose A6; SXB: sibiricaxanthone B; GTA: glomeratose A; PG3: polygalaxanthone III; TFB: tenuifoliside B; TFA: tenuifoliside A; TFC: tenuifoliside C.

The eigenvalues of the three discriminant functions were 91.9% (**F1**), 7.1% (**F2**), and 1.0% (**F3**), respectively. The distribution patterns of all of the *P. tenuifolia* samples were determined according to their quality in the plot as defined by the discriminant functions (Figure 5A), and the variations between groups were reflected by the discriminant functions **1** (91.9%) and **2** (7.1%). The use of the two most discriminating values enabled the complete separation of the *P. tenuifolia* samples into four groups (G1–G4). The plot in Figure 5B was designed by the first two discriminant functions (F1 and F2), whereas the corresponding loading plot described the concentrations of the nine components related to the separation. F1 contributed 91.9% of the variance, which provided the main separation regarding the quality among the *P. tenuifolia* samples. F1 had a strong positive correlation with DISS, sibiricose A5, and tenuifoliside B. F2 (7.1% of the variance) had a positive correlation with concentrations of sibiricose A5 and glomeratose A. The correlation between Figure 5A,B was observed as the most useful variable for discriminating the quality of all of the *P. tenuifolia* samples. The main separators among the four *P. tenuifolia* groups were the medicinal compositions of DISS, sibiricose A5, tenuifoliside B, and glomeratose A (Figure 5B). This study showed that the *P. tenuifolia* samples with different qualities were plotted in disparate spaces.

To verify the reliability of the implemented classification model, the original method and a cross-validated method were performed to calculate the classification and probability of the *P. tenuifolia* samples [32]. Table 4 summarizes the results obtained by the original, cross-validated, and DA methods. The original and cross-validated methods achieved a 100.0% correct classification of all of the *P. tenuifolia* samples. The misrepresentation or misjudgment of the case showed that the nine components were valid for discriminating each *P. tenuifolia* sample in the four groups, suggesting that the DA results were similar to the findings of HCA and radar plot analysis. Therefore, the QAMS method with chemometrics analysis is a reliable analytical strategy for evaluating the quality of *P. tenuifolia.*

## 3. Materials and Methods

### 3.1. Chemicals and Materials

Sibiricose A5, sibiricose A6, DISS, tenuifoliside A, tenuifoliside C, sibiricaxanthone B, and polygalaxanthone III were purchased from Chengdu Push Bio-Technology Co., Ltd. (Chengdu, China). Glomeratose A and tenuifoliside B were supplied by Shanghai Zeye Bio-Technology Co., Ltd. (Shanghai, China). The purity of each compound was verified to be greater than 98% by high-performance liquid chromatography (HPLC) using the peak area normalization method. The chemical structures of these compounds are shown in Figure 1. The samples of *P. tenuifolia* were collected from three provinces (Shanxi, Shaanxi, and Hebei) in China, and cultivated for three years (Supplementary Table S4). All of the herbal samples were authenticated as the root of authentic *P. tenuifolia* by Professor Yunsheng Zhao. Methanol, formic acid, and acetonitrile (HPLC grade) were obtained from Fisher Scientific Co. (Fair Lawn, NJ, USA). Purified water was purchased from Wahaha Group Co., Ltd. (Hangzhou, China).

### 3.2. Experimental Design

**Figure 6:**
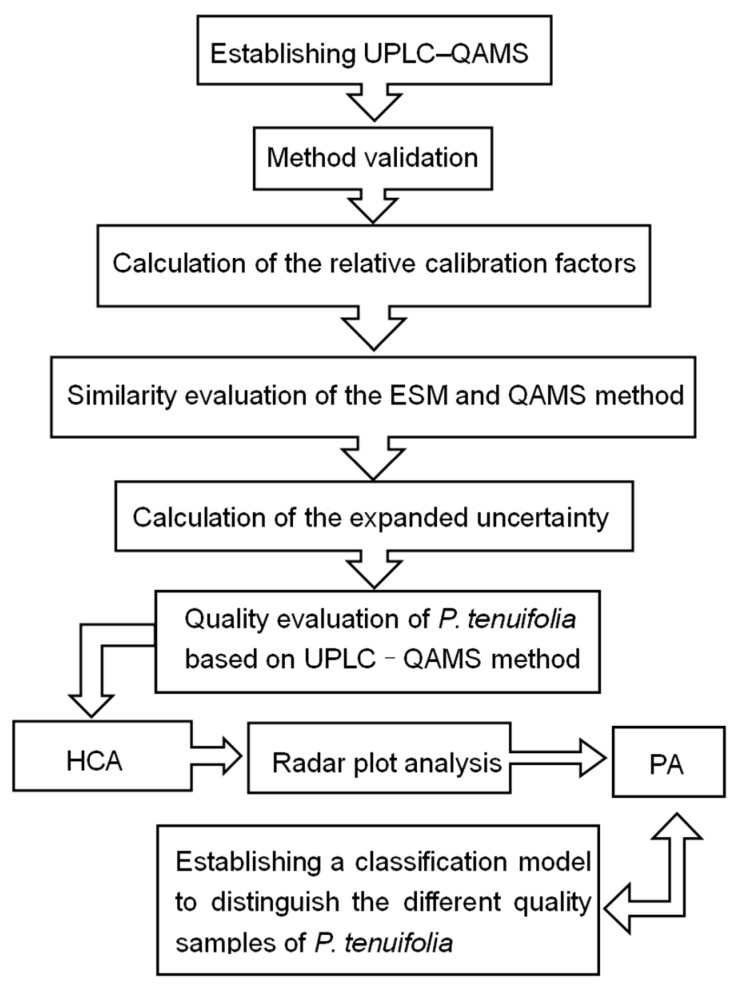
Flow diagram of the experiment.

An accessible UPLC–QAMS method was established for the quality evaluation of *P. tenuifolia*. First, UPLC fingerprints were performed to separate the index compositions from *P. tenuifolia* extracts. Second, the method validation was performed to investigate the precision stability repeatability recovery, and so on. Third, the RCFs were calculated with a series of injection volumes of the indicators, and the robustness of the RCFs were validated under different UPLC instruments, columns, column temperatures, and flow rates of the mobile phase. Fourth, the applicability and feasibility of the methodology was assessed based on the angle cosine value between the QAMS method and the ESM. Fifth, the expanded uncertainty was measured to validate the reliability of the result. Lastly, on the basis of the concentrations of the nine components, HCA, radar plot analysis, and DA were conducted to classify the 23 batches of *P. tenuifolia* with different qualities (Figure 6).

### 3.3. Chromatographic Conditions

**Figure 7:**
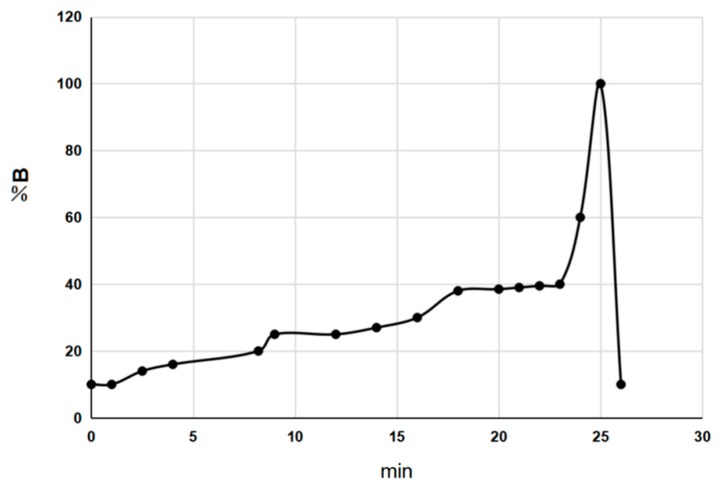
The gradient program of solvent B.

UPLC fingerprints were measured by using an ACQUITY UPLC H-Class System (Waters, Milford, MA, USA) equipped with a quaternary solvent delivery pump, an auto-sampler manager, a column compartment, and a photo diode array detector, and connected to Waters Empower 3 software. A Waters ACQUITY UPLC HSS T3 (50 mm × 2.1 mm, 1.8 μm) was used for the separation at 35 °C. The binary gradient elution system consisted of solvent A (0.1% formic acid aqueous solution) and solvent B (acetonitrile). The gradient program of solvent B was showed in Figure 7 at a flow rate of 0.3 mL·min^−1^. The detection wavelength was set at 320 nm with a sample injection volume of 1.0 μL.

### 3.4. Preparation of Sample Solutions

The dry root of *P. tenuifolia* was pulverized, and the powder was screened through a 50-mesh sieve. The powder was placed in an oven under 60 °C to a constant weight. Stock solutions were prepared by placing 1.0 g powder and 25 mL 70 *v*/*v* methanol in a 50 mL triangular flask. The solutions were then sonicated for 40 min, and allowed to cool to room temperature. Then, 70 *v*/*v* methanol was complemented for weightlessness. All of the sample solutions were filtered through a 0.22 μm millipore filter prior to UPLC analysis. The stock and working solutions of the standards were prepared in dark brown volumetric flasks and stored at 4 °C.

### 3.5. Preparation of Standard Solutions

Nine stock solutions were prepared by dissolving each reference standard in methanol. The working solution of all of the standards was mixed with nine stock solutions immediately before analyses with the required concentrations: sibiricose A5, 67.07 mg·L^−1^; sibiricose A6, 49.85 mg·L^−1^; glomeratose A, 56.82 mg·L^−1^; DISS, 342.02 mg·L^−1^; tenuifoliside A, 216.92 mg·L^−1^; tenuifoliside B, 57.23 mg·L^−1^; tenuifoliside C, 90.30 mg·L^−1^; sibiricaxanthone B, 50.59 mg·L^−1^; polygalaxanthone III, 24.06 mg·L^−1^. The standards solutions were prepared in dark brown volumetric flasks and stored at 4 °C.

### 3.6. Quantitative Analysis of Multi-Components by Single Marker

The QAMS method was applied to the quality control of *P. tenuifolia* by using RCFs that were calculated based on each component, which was proportional to the detector within a certain range [33]. A suitable reference component was selected. The selected reference component should possess the following characteristics: low cost, high stability, high concentration, easy to obtain, significant pharmacological activities, and easily separated under conventional chromatographic conditions. In this study, DISS was chosen as a standard for the quantitative analysis of other components, including sibiricose A5, sibiricose A6, glomeratose A, tenuifoliside A, tenuifoliside B, tenuifoliside C, sibiricaxanthone B, and polygalaxanthone III. The RCF can be calculated as follows:

(5) $$f_{si}=\frac{f_{s}}{f_{i}}=\frac{A_{s}/C_{s}}{A_{i}/C_{i}},$$

where *A_s_* is the peak area of the internal reference substance, *C_s_* is the concentration of the internal reference substance, *A_i_* is the peak area of sample *i*, and *C_i_* is the concentration of sample *i*. According to Equation (5), the concentration of component *i* (*C_i_*) can be calculated by using Equation (6).

(6) $$C_{i}=f_{si}\times C_{s}\times \frac{A_{i}}{A_{s}},$$

### 3.7. Assessment of the ESM and the QAMS Method 

The cosine ratio value (*C_ir_*) method is an algorithm that is generally used to assess the similarity of the QAMS method and the ESM. *C_ir_* is a vector that calculates the angle between the two groups of variables to measure their similarity in Euclidian geometry, and it is defined in Equation (7) [34]. *C_ir_* was calculated by using Microsoft Excel 2003 software in this study.

(7) $$C_{ir}=\frac{\sum_{k=1}^{n}X_{ik}\cdot X_{rk}}{\sqrt{\left(\sum_{k=1}^{n}X_{ik}^{2})(\sum_{k=1}^{n}X_{rk}^{2}\right)}},$$

where *X_ik_* is the value of compound *k* in sample *i* in the ESM, and *X_rk_* is the value of compound *k* of the same sample in the QAMS method.

### 3.8. Evaluation and Calculation of Expanded Uncertainty 

Scientific research is based on analytical measurements; uncertainty is a basic characteristic of any measurement, and helps to assure the reliability of an analyst’s work. Uncertainty expresses the doubt inherent to any measurement process concerning a given measurement, such as X ± *U* (*k* = y), where *U* is the expanded measurement uncertainty, and *k* is the coverage factor. Typically, a coverage factor of two is applied, ensuring a confidence interval of 95%. The expanded uncertainty measurement result can be described by the formula:

(8) $$U=kc\sqrt{{\left(u_{r(\mathrm{sample})}\right)}^{2}+{\left(u_{r(\mathrm{cal})}\right)}^{2}+{\left(u_{r(\mathrm{true})}\right)}^{2}+{\left(u_{r(\mathrm{rep})}\right)}^{2}+{\left(u_{r(\mathrm{LOD})}\right)}^{2}},$$

where: *c*—average concentration of the analyte, *u_r_*_(sample)_—relative standard uncertainty of the sample mass determination, *u_r_*_(cal)_—relative standard uncertainty of the calibration step, *u_r_*_(true)_—relative standard uncertainty of recovery determination, *u_r_*_(rep)_—relative standard uncertainty of repeatability, and *u_r_*_(LOD)_—relative standard uncertainty of LOD determination [35]. The uncertainty associated with the estimate of recovery in each sample was calculated as Barwick and Konieczka reported [36,37].

(9) $$u_{r(\mathrm{cal})}=\frac{{\mathrm{SD}}_{\mathrm{RRF}}}{\sqrt{n}},$$

(10) $$u_{r(\mathrm{rep})}=\frac{{\mathrm{SD}}_{\mathrm{results}}}{\sqrt{n}},$$

(11) $$u_{r(\mathrm{LOD})}=\frac{\mathrm{LOD}}{c_{\det}},$$

where: SD_RRF_—standard deviation of the measurement series, *n*—the number of repetitions, SD_results_—standard deviation of the results, and *c*_det_—the concentrations of the results [37].

### 3.9. Data Analyses 

HCA is a chemical pattern recognition and classification evaluation method that is used to classify objects (samples) into clusters, such that each object is similar to the others within a cluster, but different from those in other clusters with respect to a predetermined selection criterion [38]. Radial plots have been introduced as descriptive tools for multivariate data. In general, the common feature of radial plots is that they are applied with a relatively small number of independent variables, and that they have a series of spokes or rays projecting from a central point, with each ray representing a different variable label. In such diagrams, the values of the variables are encoded into the lengths of the rays. The plotted values are then connected by lines to form an enclosed figure [39]. Dominant perceptual properties often include the size and shape of the resulting figure [40]. DA can be used to provide a statistical classification of samples sharing common properties and build a predictive model of the group membership on the basis of the observed characteristics in each case. This procedure generates a discriminant function—or a set of discriminant functions—from the samples with a known membership on the basis of the linear combinations of the predictor variables, and provides the best discrimination among the groups. The functions can then be applied to discriminate and classify new cases with unknown group membership on the basis of the measurements of the predictor variables. Such a function represents a surface that divides the data space into regions [41]. HCA and DA were conducted by using SPSS (International Business Machines Corporation, New York, NY, USA) 18.0 statistical software. Radar plot analysis was manipulated by using Microsoft Excel 2003.

## 4. Conclusions

In this study, QAMS was developed for the simultaneous quantitative analysis of nine components in 23 batches of *P. tenuifolia* by UPLC. DISS was chosen as the internal reference substance. The RCFs between DISS and the other eight components were investigated with QAMS calculations under different instruments, columns, column temperatures, and flow rates of the mobile phase. For comparison, the concentrations of the nine components were also determined by a conventional ESM. Finally, the angle cosine value was calculated, and the results revealed that no significant difference existed between the newly established QAMS method and the traditional ESM. *P. tenuifolia*’s main traditional efficacy is that it tranquilizes the mind and promotes intelligence, which is mainly related to the oligosaccharide esters in *P. tenuifolia*. Seven oligosaccharide concentrations were determined simultaneously in this experiment by QAMS–UPLC, which reflected more information than the one oligosaccharide ester (DISS) determined in Chinese pharmacopoeia. Xanthones are the anti-inflammatory constituents of *P. tenuifolia*. Two xanthones were measured in this study, whereas the Chinese pharmacopoeia requires only one to be tested. Saponins are considered important active ingredients of expectorants in *P. tenuifolia*. However, compared with oligosaccharide esters and xanthones, which are extracted with alcohol and detected in 320 nm, the extraction (alkaline hydrolysis) and determination (in 210 nm) of saponins are significantly different. Therefore, the saponins were not measured in this experiment [42]. On the other hand, the UPLC–QAMS measurement took only 26 min in this study, and saved about 40 min compared with the general HPLC fingerprinting study (70 min) [43]. Meanwhile, only one single reference substance was used in this experiment, which greatly reduced the cost of testing and was easily used for the scientific research and supervised application. However, the ESM required multiple reference standards and analyzed only one component at a time. This study improved the quality standard by establishing UPLC–QAMS, which is useful for providing an efficient and feasible quality assessment of *P. tenuifolia*. QAMS combined with HCA, radar plot analysis, and DA is a potential method that can comprehensively and effectively estimate the quality of *P. tenuifolia*, and thus has promising wide applications in the quality control of *P. tenuifolia*.

## Figures and Tables

**Table 1:** Calibration curves for the nine compounds determined. LOD: limit of detection, LOQ: limit of quantification; MDL: method detection limit; MQL: method quantitation limit.

Reference Substance	Rt	Regression Equation	R^2^	Linear Range	LOD	LOQ	MDL	MQL
	min			μg·mL^−1^	μg·mL^−1^	μg·mL^−1^	μg·mL^−1^	μg·mL^−1^
sibiricose A5	1.99	y = 6.606 × 10^6^x + 1683.6	0.9998	6.71~93.90	0.14	0.45	0.08	0.26
sibiricose A6	2.33	y = 6.541 × 10^6^x − 2614	0.9992	4.99~69.79	0.11	0.36	0.06	0.2
sibiricaxanthone B	4.72	y = 4.412 × 10^6^x − 545.72	0.9999	5.06~70.83	0.13	0.43	0.06	0.19
glomeratose A	5.11	y = 5.450 × 10^6^x − 839.47	0.9999	5.68~79.55	0.1	0.33	0.05	0.17
polygalaxanthone III	5.44	y = 6.332 × 10^6^x − 2350.7	0.9999	2.41~33.68	0.06	0.2	0.03	0.1
tenuifoliside B	6.19	y = 4.907 × 10^6^x − 1748.2	0.9999	5.72~80.12	0.2	0.66	0.08	0.26
DISS	8.69	y = 9.222 × 10^6^x – 24939	0.9999	34.20~478.83	0.06	0.22	0.04	0.12
tenuifoliside A	10.11	y = 4.137 × 10^6^x − 6735.5	0.9999	21.69~303.70	0.14	0.45	0.06	0.18
tenuifoliside C	11.54	y = 9.104 × 10^6^x − 5511.8	0.9999	9.03~126.42	0.06	0.21	0.03	0.11

**Table 2:** Intra-day and inter-day precisions, stability, repeatability, and recovery for the nine components (*n* = 6). RSD: relative standard deviation.

Compounds	Intra-Day RSD/%	Inter-Day RSD/%	Intermediate RSD/%	Stability RSD/%	Repeatability RSD/%	Recovery RSD/%
sibiricose A5	0.30	0.42	1.70	0.22	1.78	97.33, 1.05
sibiricose A6	0.19	0.40	0.37	0.57	1.61	96.75, 2.36
sibiricaxanthone B	0.33	0.64	1.55	0.38	1.44	98.11, 1.03
glomeratose A	0.23	0.59	1.02	0.27	1.72	97.61, 1.03
polygalaxanthone III	0.37	0.50	1.40	0.29	1.34	97.93, 1.19
tenuifoliside B	0.69	0.48	0.62	1.40	1.76	101.90, 1.48
DISS	0.34	0.44	0.00	0.11	1.61	98.03, 1.08
Tenuifoliside A	0.60	0.65	0.75	0.21	3.27	97.22, 3.47
tenuifoliside C	0.83	1.07	1.85	2.86	1.68	95.48, 2.77

**Table 3:** The concentrations of nine components of *P. tenuifolia* by the external standard method (ESM) and the quantitative analysis of multi-components by a single marker (QAMS) method (mg/g, *n* = 3) ^1^.

No.	DISS	Sibiricose A5		Sibiricose A6		Sibiricaxanthone B		Glomeratose A		Polygalaxanthone III		Tenuifoliside B		Tenuifoliside A		Tenuifoliside C	
	ESM	QAMS	ESM	QAMS	ESM	QAMS	ESM	QAMS	ESM	QAMS	ESM	QAMS	ESM	QAMS	ESM	QASM	ESM
S1	6.23 ± 0.25	1.45 ± 0.09	1.44 ± 0.07	1.08 ± 0.08	1.08 ± 0.07	0.96 ± 0.06	0.97 ± 0.05	0.64 ± 0.04	0.65 ± 0.03	0.46 ± 0.03	0.46 ± 0.02	0.48 ± 0.03	0.48 ± 0.03	4.03 ± 0.38	4.06 ± 0.35	1.82 ± 0.15	1.84 ± 0.13
S2	7.15 ± 0.29	1.32 ± 0.08	1.31 ± 0.07	1.30 ± 0.10	1.30 ± 0.09	0.86 ± 0.05	0.87 ± 0.04	0.77 ± 0.05	0.77 ± 0.04	0.53 ± 0.04	0.54 ± 0.03	0.44 ± 0.03	0.45 ± 0.03	3.08 ± 0.29	3.12 ± 0.27	1.82 ± 0.15	1.84 ± 0.13
S3	3.55 ± 0.16	0.57 ± 0.04	0.56 ± 0.03	0.52 ± 0.04	0.52 ± 0.04	0.66 ± 0.05	0.65 ± 0.04	0.88 ± 0.06	0.88 ± 0.04	0.55 ± 0.04	0.55 ± 0.03	0.31 ± 0.02	0.32 ± 0.02	3.68 ± 0.33	3.67 ± 0.29	1.88 ± 0.15	1.87 ± 0.12
S4	4.89 ± 0.23	0.69 ± 0.05	0.68 ± 0.03	0.84 ± 0.07	0.84 ± 0.05	0.78 ± 0.06	0.78 ± 0.04	0.75 ± 0.05	0.75 ± 0.04	0.51 ± 0.04	0.51 ± 0.03	0.43 ± 0.03	0.44 ± 0.02	2.78 ± 0.27	2.81 ± 0.24	1.95 ± 0.16	1.96 ± 0.13
S5	5.00 ± 0.23	1.03 ± 0.07	1.02 ± 0.05	1.03 ± 0.08	1.03 ± 0.07	0.70 ± 0.05	0.71 ± 0.04	0.69 ± 0.05	0.70 ± 0.04	0.50 ± 0.04	0.51 ± 0.03	0.32 ± 0.02	0.32 ± 0.02	2.62 ± 0.25	2.65 ± 0.22	1.51 ± 0.13	1.52 ± 0.11
S6	6.95 ± 0.28	2.15 ± 0.15	2.13 ± 0.11	1.44 ± 0.12	1.44 ± 0.11	1.08 ± 0.07	1.09 ± 0.05	0.77 ± 0.05	0.78 ± 0.04	0.66 ± 0.04	0.66 ± 0.03	0.49 ± 0.03	0.49 ± 0.03	4.32 ± 0.38	4.36 ± 0.35	2.40 ± 0.20	2.41 ± 0.18
S7	3.98 ± 0.16	1.33 ± 0.09	1.30 ± 0.06	1.04 ± 0.08	1.04 ± 0.07	0.81 ± 0.05	0.80 ± 0.04	0.88 ± 0.06	0.88 ± 0.04	0.53 ± 0.04	0.53 ± 0.03	0.31 ± 0.02	0.31 ± 0.02	4.32 ± 0.41	4.31 ± 0.37	1.82 ± 0.15	1.82 ± 0.13
S8	5.16 ± 0.19	0.98 ± 0.06	0.97 ± 0.05	1.00 ± 0.07	1.00 ± 0.06	0.76 ± 0.05	0.77 ± 0.04	0.78 ± 0.05	0.78 ± 0.04	0.51 ± 0.03	0.51 ± 0.03	0.43 ± 0.03	0.44 ± 0.02	2.65 ± 0.23	2.68 ± 0.21	1.17 ± 0.09	1.18 ± 0.08
S9	7.01 ± 0.29	1.38 ± 0.10	1.38 ± 0.08	1.38 ± 0.11	1.38 ± 0.09	0.65 ± 0.05	0.66 ± 0.04	0.81 ± 0.06	0.82 ± 0.05	0.49 ± 0.04	0.50 ± 0.03	0.55 ± 0.04	0.55 ± 0.04	2.39 ± 0.23	2.43 ± 0.21	2.00 ± 0.17	2.02 ± 0.15
S10	6.45 ± 0.31	1.87 ± 0.13	1.86 ± 0.09	1.77 ± 0.15	1.76 ± 0.12	1.36 ± 0.10	1.36 ± 0.08	1.11 ± 0.07	1.12 ± 0.05	0.73 ± 0.05	0.73 ± 0.04	0.63 ± 0.05	0.64 ± 0.04	5.09 ± 0.48	5.12 ± 0.42	2.29 ± 0.19	2.30 ± 0.16
S11	5.89 ± 0.24	0.80 ± 0.05	0.79 ± 0.04	0.91 ± 0.07	0.91 ± 0.06	0.86 ± 0.05	0.86 ± 0.04	0.96 ± 0.06	0.97 ± 0.05	0.54 ± 0.04	0.55 ± 0.03	0.54 ± 0.04	0.55 ± 0.03	3.33 ± 0.32	3.36 ± 0.29	1.43 ± 0.11	1.44 ± 0.10
S12	5.46 ± 0.25	1.91 ± 0.12	1.89 ± 0.09	1.37 ± 0.11	1.36 ± 0.09	1.16 ± 0.08	1.17 ± 0.05	0.86 ± 0.05	0.86 ± 0.04	0.88 ± 0.06	0.88 ± 0.04	0.93 ± 0.06	0.93 ± 0.05	5.17 ± 0.47	5.19 ± 0.41	2.01 ± 0.16	2.02 ± 0.13
S13	6.59 ± 0.27	1.45 ± 0.09	1.44 ± 0.07	1.59 ± 0.12	1.59 ± 0.10	0.83 ± 0.06	0.84 ± 0.05	0.72 ± 0.04	0.73 ± 0.03	0.55 ± 0.04	0.56 ± 0.03	0.61 ± 0.04	0.61 ± 0.04	4.20 ± 0.40	4.24 ± 0.36	2.14 ± 0.17	2.16 ± 0.14
S14	4.96 ± 0.19	0.73 ± 0.05	0.71 ± 0.04	0.85 ± 0.07	0.85 ± 0.06	0.74 ± 0.04	0.74 ± 0.04	0.68 ± 0.04	0.68 ± 0.03	0.48 ± 0.03	0.49 ± 0.03	0.41 ± 0.03	0.41 ± 0.02	2.69 ± 0.25	2.71 ± 0.23	1.31 ± 0.11	1.32 ± 0.10
S15	3.74 ± 0.16	0.79 ± 0.05	0.78 ± 0.04	0.75 ± 0.06	0.75 ± 0.05	0.77 ± 0.05	0.76 ± 0.04	0.94 ± 0.07	0.94 ± 0.06	0.48 ± 0.03	0.48 ± 0.02	0.33 ± 0.02	0.33 ± 0.02	3.74 ± 0.34	3.73 ± 0.30	1.50 ± 0.13	1.50 ± 0.11
S16	6.70 ± 0.30	2.22 ± 0.14	2.21 ± 0.10	1.48 ± 0.12	1.47 ± 0.10	1.11 ± 0.07	1.12 ± 0.06	0.80 ± 0.05	0.81 ± 0.04	0.67 ± 0.05	0.67 ± 0.04	0.53 ± 0.04	0.54 ± 0.04	4.51 ± 0.42	4.54 ± 0.37	2.30 ± 0.18	2.32 ± 0.15
S17	4.19 ± 0.21	0.96 ± 0.07	0.94 ± 0.04	0.94 ± 0.08	0.94 ± 0.06	0.71 ± 0.05	0.71 ± 0.03	0.78 ± 0.06	0.78 ± 0.04	0.46 ± 0.03	0.46 ± 0.02	0.33 ± 0.03	0.34 ± 0.02	2.73 ± 0.27	2.75 ± 0.24	1.38 ± 0.12	1.38 ± 0.09
S18	3.56 ± 0.17	1.02 ± 0.07	1.00 ± 0.05	0.70 ± 0.06	0.70 ± 0.05	0.69 ± 0.05	0.69 ± 0.03	0.69 ± 0.05	0.69 ± 0.04	0.53 ± 0.04	0.53 ± 0.03	0.31 ± 0.03	0.32 ± 0.02	3.15 ± 0.30	3.15 ± 0.26	1.39 ± 0.12	1.39 ± 0.10
S19	5.20 ± 0.25	1.19 ± 0.08	1.18 ± 0.06	1.11 ± 0.10	1.11 ± 0.08	0.73 ± 0.05	0.73 ± 0.04	0.62 ± 0.04	0.63 ± 0.03	0.47 ± 0.03	0.47 ± 0.03	0.42 ± 0.03	0.44 ± 0.03	2.47 ± 0.24	2.50 ± 0.21	1.34 ± 0.11	1.35 ± 0.09
S20	6.11 ± 0.23	1.60 ± 0.10	1.58 ± 0.08	1.10 ± 0.08	1.10 ± 0.07	0.93 ± 0.05	0.94 ± 0.04	0.71 ± 0.04	0.72 ± 0.03	0.74 ± 0.05	0.74 ± 0.04	0.46 ± 0.03	0.48 ± 0.03	3.49 ± 0.33	3.53 ± 0.31	2.26 ± 0.17	2.27 ± 0.15
S21	5.71 ± 0.21	1.16 ± 0.07	1.15 ± 0.05	1.22 ± 0.09	1.22 ± 0.08	1.08 ± 0.06	1.09 ± 0.05	0.78 ± 0.05	0.78 ± 0.04	0.77 ± 0.05	0.77 ± 0.04	0.85 ± 0.05	0.88 ± 0.04	4.08 ± 0.35	4.10 ± 0.32	1.32 ± 0.10	1.33 ± 0.09
S22	7.04 ± 0.29	1.66 ± 0.11	1.65 ± 0.08	1.48 ± 0.12	1.48 ± 0.10	0.89 ± 0.06	0.89 ± 0.04	0.76 ± 0.05	0.77 ± 0.04	0.61 ± 0.04	0.61 ± 0.03	0.35 ± 0.03	0.35 ± 0.02	3.43 ± 0.33	3.47 ± 0.30	1.66 ± 0.13	1.67 ± 0.12
S23	6.35 ± 0.29	1.87 ± 0.12	1.86 ± 0.08	1.33 ± 0.11	1.33 ± 0.09	0.86 ± 0.07	0.86 ± 0.05	0.70 ± 0.05	0.70 ± 0.03	0.61 ± 0.05	0.62 ± 0.04	0.40 ± 0.03	0.41 ± 0.02	3.11 ± 0.29	3.14 ± 0.26	1.83 ± 0.15	1.85 ± 0.12
Mean	5.523	1.285	1.271	1.140	1.139	0.867	0.872	0.786	0.789	0.577	0.578	0.471	0.480	3.524	3.548	1.762	1.772
*C_ir_*		0.999990		0.999998		0.999994		0.999990		0.999993		0.999920		0.999990		0.999993	

^1^ Mean ± expanded uncertainty. *k* = 2.

**Table 4:** Observations of the original and cross-validation results together with the classification of *P. tenuifolia* samples using the discriminant analysis model.

**Group**	**Assigned Origin for All of the Cultivated *P. tenuifolia* Samples with Nine Components**					
	**G1**	**G2**	**G3**	**G4**	**Total**	**Original Correct (%)**
G1	4	0	0	0	4	100.0
G2	0	9	0	0	9	100.0
G3	0	0	5	0	5	100.0
G4	0	0	0	5	5	100.0
Total	4	9	5	5	23	100.0
**Group**	**G1**	**G2**	**G3**	**G4**	**Total**	**Cross-Validation Correct (%)**
G1	4	0	0	0	4	100.0
G2	0	9	0	0	9	100.0
G3	0	0	5	0	5	100.0
G4	0	0	0	5	5	100.0
Total	4	9	5	5	23	100.0

## Supplementary Materials

The following are available online. Tables S1–S4.

## Abbreviations

The following abbreviations are used in this manuscript.

QAMS	Quantitative analysis of multi-components by a single marker
TCM	Traditional Chinese medicine
SDMC	Simultaneous determination of multi-components
UPLC	Ultra-performance liquid chromatography
HPLC	High-performance liquid chromatography
ESM	External standard method
DISS	3,6′-disinapoyl sucrose
RCFs	Relative calibration factors
HCA	Hierarchical cluster analysis
DA	Discriminant analysis
LOQ	Limit of quantification
LOD	Limit of detection
MDL	Method detection limit
MQL	Method quantitation limit
QA	Quality assurance
QC	Quality control
SD	Standard deviation

## References

[1] Xie P.S., Leung A.Y., Xie P.S., van Beek T.A. (2009). Understanding the traditional aspect of Chinese medicine in order to achieve meaningful quality control of Chinese materia medica. J. Chromatogr. A.

[2] Liu E.H., Qi L.W., Li K., Chu C., Li P. (2010). Recent advances in quality control of traditional Chinese medicines. Comb. Chem. High Throughput Screen..

[3] Shi Z., Liu Z., Liu C., Wu M., Su H., Ma X., Zang Y., Wang J., Zhao Y., Xiao X. (2016). Spectrum-Effect Relationships Between Chemical Fingerprints and Antibacterial Effects of Lonicerae Japonicae Flos and Lonicerae Flos Base on UPLC and Microcalorimetry. Front. Pharmacol..

[4] Jakimska A., Kot-Wasik A., Namieśnik J. (2014). The Current State-of-the-Art in the Determination of Pharmaceutical Residues in Environmental Matrices Using Hyphenated Techniques. Crit. Rev. Anal. Chem..

[5] Kumar A., Saini G., Nair A., Sharma R. (2012). UPLC: A preeminent technique in pharmaceutical analysis. Acta Pol. Pharm..

[6] Sun Z., Zhao Y., Liu T., Sun X., Li R., Zhang P., Xiao X. (2013). Spectrum-effect relationships between UPLC fingerprints and bioactivities of five *Aconitum* L. plants. Thermochim. Acta.

[7] Kong W.J., Zhao Y.L., Xiao X.H., Wang J.B., Li H.B., Li Z.L., Jin C., Liu Y. (2009). Spectrum-effect relationships between ultra performance liquid chromatography fingerprints and anti-bacterial activities of *Rhizoma coptidis*. Anal. Chim. Acta.

[8] Nassar A.F., Wu T., Nassar S.F., Wisnewski A.V. (2016). UPLC-MS for metabolomics: A giant step forward in support of pharmaceutical research. Drug Discov. Today.

[9] Jin J.Q., Ma J.Q., Ma C.L., Yao M.Z., Chen L. (2014). Determination of catechin concentration in representative Chinese tea germplasms. J. Agric. Food Chem..

[10] Wang C.Q., Jia X.H., Zhu S., Komatsu K., Wang X., Cai S.Q. (2015). A systematic study on the influencing parameters and improvement of quantitative analysis of multi-component with single marker method using notoginseng as research subject. Talanta.

[11] Cui L., Zhang Y., Shao W., Gao D. (2016). Analysis of the HPLC fingerprint and QAMS from *Pyrrosia* species. Ind. Crops Prod..

[12] Du Y., Li Q., Liu J., Yin Y., Bi K. (2014). Combinative method using multi-components quantitation by single reference standard and HPLC fingerprint for comprehensive evaluation of *Rhodiola crenulata* H.Ohba. Anal. Methods.

[13] Li S.P., Qiao C.F., Chen Y.W., Zhao J., Cui X.M., Zhang Q.W., Liu X.M., Hu D.J. (2013). A novel strategy with standardized reference extract qualification and single compound quantitative evaluation for quality control of *Panax notoginseng* used as a functional food. J. Chromatogr. A.

[14] Tang W., Eisenbrand G. (1992). Chinese Drugs of Plant Origin.

[15] Dong X.Z., Huang C.L., Yu B.Y., Hu Y., Mu L.H., Liu P. (2014). Effect of tenuifoliside A isolated from *Polygala tenuifolia* on the ERK and PI3K pathways in C6 glioma cells. Phytomedicine.

[16] Jiang H., Liu T., Li L., Zhao Y., Pei L., Zhao J. (2016). Predicting the Potential Distribution of *Polygala tenuifolia* Willd. under Climate Change in China. PLoS ONE.

[17] Nagajyothi P.C., Sang J.C., Yang I.J., Sreekanth T.V.M., Kim K.J., Shin H.M. (2015). Antioxidant and anti-inflammatory activities of zinc oxide nanoparticles synthesized using *Polygala tenuifolia* root extract. J. Photochem. Photobiol. B Biol..

[18] Zhang F., Li X., Li Z., Xu X., Peng B., Qin X., Du G. (2014). UPLC/Q-TOF MS-Based Metabolomics and qRT-PCR in Enzyme Gene Screening with Key Role in Triterpenoid Saponin Biosynthesis of *Polygala tenuifolia*. PLoS ONE.

[19] Zhang F., Song X., Li L., Wang J., Lin L., Li C., Li H., Lv Y., Jin Y., Liu Y. (2015). *Polygala tenuifolia* polysaccharide PTP induced apoptosis in ovarian cancer cells via a mitochondrial pathway. Tumor Biol..

[20] Wang H., Gao J., Zhu D., Yu B. (2007). Quality evaluation of Polygala japonica through simultaneous determination of six bioactive triterpenoid saponins by HPLC-ELSD. J. Pharm. Biomed. Anal..

[21] Yang X.D., Zhang L.J., Liang B., Li Zhen X.U., Yang S.L. (2002). Oligosaccharide esters isolated from plants of Polygalaceae. Chin. Tradit. Herb. Drugs.

[22] Chen S.L., Lin L.L., Chen S.B., Yang D.J., Yang J.S., Xiao P.G. (2005). Quantitative Determination of Nine Xanthones in *Polygala caudata* and Fingerprinting of *Polygala* L. by HPLC. J. Liq. Chromatogr. Relat. Technol..

[23] Sun X.L., Ito H., Masuoka T., Kamei C., Hatano T. (2007). Effect of *Polygala tenuifolia* root extract on scopolamine-induced impairment of rat spatial cognition in an eight-arm radial maze task. Biol. Pharm. Bull..

[24] Lin C.N., Liou S.J., Lee T.H., Chuang Y.C., Won S.J. (1996). Xanthone derivatives as potential anti-cancer drugs. J. Pharm. Pharmacol..

[25] Chen L.G., Yang L.L., Wang C.C. (2008). Anti-inflammatory activity of mangostins from *Garcinia mangostana*. Food Chem. Toxicol. Int. J. Publ. Br. Ind. Biol. Res. Assoc..

[26] Lin C.N., Chung M.I., Liou S.J., Lee T.H., Wang J.P. (1996). Synthesis and anti-inflammatory effects of xanthone derivatives. J. Pharm. Pharmacol..

[27] Suksamrarn S., Suwannapoch N., Phakhodee W., Thanuhiranlert J., Ratananukul P., Chimnoi N., Suksamrarn A. (2003). Antimycobacterial activity of prenylated xanthones from the fruits of *Garcinia mangostana*. Chem. Pharm. Bull..

[28] Sun X.P., Li S.D., Shi Z., Li T.F., Pan R.L., Chang Q., Qin C., Liu X.M. (2013). Antidepressant-like effects and memory enhancement of a herbal formula in mice exposed to chronic mild stress. Sci. Bull..

[29] Dang H., Chen Y., Liu X., Wang Q., Wang L., Jia W., Wang Y. (2009). Antidepressant effects of ginseng total saponins in the forced swimming test and chronic mild stress models of depression. Prog. Neuropsychopharmacol. Biol. Psychiatry.

[30] Guo C., Wang M., Hui X., Huai B., Feng W., Pan G., Liao X., Liu Y. (2016). Development of a modified QuEChERS method for the determination of veterinary antibiotics in swine manure by liquid chromatography tandem mass spectrometry. J. Chromatogr. B.

[31] Qing L.S., Xue Y., Deng W.L., Liao X., Xu X.M., Li B.G., Liu Y.M. (2011). Ligand fishing with functionalized magnetic nanoparticles coupled with mass spectrometry for herbal medicine analysis: Ligand fishing for herbal medicine analysis. Anal. Bioanal. Chem..

[32] Cheajesadagul P., Arnaudguilhem C., Shiowatana J., Siripinyanond A., Szpunar J. (2013). Discrimination of geographical origin of rice based on multi-element fingerprinting by high resolution inductively coupled plasma mass spectrometry. Food Chem..

[33] Gao H., Song Z., Wang Z., Qian Z., Zhang Q. (2012). Overview on quantitative analysis of multi-components by single-marker. China J. Chin. Mater. Med..

[34] Xie J., Li J., Liang J., Luo P., Qing L.S., Ding L.S. (2016). Determination of concentrations of Catechins in Oolong Teas by Quantitative Analysis of Multi-components Via a Single Marker (QAMS) Method. Food Anal. Methods.

[35] International Organisation for Standardisation (1993). Guide to the Expression of Uncertainty in Measurement (GUM).

[36] Barwick V.J., Ellison S.L.R. (1999). Measurement uncertainty: Approaches to the evaluation of uncertainties associated with recovery†. Analyst.

[37] Konieczka P., Namieśnik J. (2010). Estimating uncertainty in analytical procedures based on chromatographic techniques. J. Chromatogr. A.

[38] Kannel P.R., Lee S., Kanel S.R., Khan S.P. (2007). Chemometric application in classification and assessment of monitoring locations of an urban river system. Anal. Chim. Acta.

[39] Saary M.J. (2008). Radar plots: A useful way for presenting multivariate health care data. J. Clin. Epidemiol..

[40] Friendly M., Kwan E. (2003). Effect ordering for data displays. Comput. Stat Data Anal..

[41] Shi X.M., Zhang J.S., Tang Q.J., Yang Y., Hao R.X., Pan Y.J. (2008). Fingerprint analysis of Lingzhi (*Ganoderma*) strains by high-performance liquid chromatography coupled with chemometric methods. World J. Microbiol. Biotechnol..

[42] National Pharmacopoeia Committee (2015). Pharmacopoeia of People’s Republic of China, Part 1.

[43] Zhao Y.S., Liu X., Mao F.Y., Tian H.L., Wan D.G. (2014). Study on quality assessment of Polygalae Radix based on HPLC-DAD fingerprint. Zhongguo Zhong Yao Za Zhi.

