# Supplementary material for: UPLC Quantitative Analysis of Multi-Components by Single Marker and Quality Evaluation of Polygala tenuifolia Wild. Extracts
Source: Molecules. 2017 Dec 20;22(12):2276. doi: 10.3390/molecules22122276 (PMC6149966; doi:10.3390/molecules22122276)
Supplement: Supplementary file 1 [file molecules-22-02276-s001.pdf]

Table 1. Relative correction factors (RCFs) of eight components to DISS in *P. tenuifolia* (n=3)[1].

Injection volume/μL	*f*G/A	*f*G/B	*f*G/C	*f*G/D	*f*G/E	*f*G/F	*f*G/H	*f*G/I
0.1	1.318	1.406	1.968	1.615	1.511	1.815	2.178	0.981
0.2	1.324	1.392	1.973	1.619	1.489	1.804	2.143	0.973
0.4	1.375	1.429	2.049	1.665	1.484	1.865	2.207	1.005
0.6	1.374	1.420	2.046	1.658	1.471	1.854	2.201	1.002
0.8	1.387	1.413	2.065	1.669	1.468	1.863	2.208	1.004
1	1.387	1.398	2.058	1.665	1.454	1.855	2.199	0.999
1.2	1.394	1.384	2.056	1.664	1.448	1.852	2.196	0.997
1.4	1.408	1.418	2.050	1.661	1.471	1.849	2.195	0.997
Mean	1.371	1.408	2.033	1.652	1.475	1.845	2.191	0.995
RSD/%	2.384	1.087	1.919	1.317	1.355	1.216	0.973	1.171

[1] Note: A: sibiricose A5; B: sibiricose A6; C: sibiricaxanthone B; D: glomeratose A; E: polygalaxanthone III; F: tenuifolisideB; G: 3,6′-disinapoyl sucrose (DISS); H: tenuifolisideA; I: tenuifolisideC.

Table 2. Relative correction factors (RCFs) determined by different instruments and columns (n=3)[2].

Instrument	Column	*f*G/A	*f*G/B	*f*G/C	*f*G/D	*f*G/E	*f*G/F	*f*G/H	*f*G/I
Waters ACQUITY	Waters TSS-C18	1.315	1.393	2.090	1.608	1.453	1.811	2.146	1.023
	Waters BEH-C18	1.316	1.342	2.065	1.589	1.501	1.772	2.200	1.100
Waters ACQUITY H-Class	Waters TSS-C18	1.387	1.398	2.058	1.665	1.454	1.855	2.199	0.999
	Waters BEH-C18	1.400	1.313	1.993	1.641	1.479	1.859	2.248	0.992
Mean		1.354	1.361	2.051	1.626	1.472	1.824	2.198	1.028
RSD/%		3.353	3.018	1.998	2.065	1.535	2.268	1.910	4.853

[2] Note: A: sibiricose A5; B: sibiricose A6; C: sibiricaxanthone B; D: glomeratose A; E: polygalaxanthone III; F: tenuifolisideB; G: 3,6′-disinapoyl sucrose (DISS); H: tenuifolisideA; I: tenuifolisideC.

Table 3. Relative correction factors (RCFs) determined by different column temperatures and mobile phase flow rates (n=3)[3].

	Column temperature (℃)				Flow rates (mL·min-1)			
	**30**	**35**	**40**	**RSD%**	**0.28**	**0.30**	**0.32**	**RSD%**
fG/A	1.374	1.387	1.400	0.949	1.419	1.387	1.389	1.267

fG/B	1.403	1.398	1.404	0.232	1.422	1.398	1.405	0.875
fG/C	2.040	2.058	2.022	0.878	2.085	2.058	2.031	1.301
fG/D	1.648	1.665	1.701	1.615	1.713	1.665	1.676	1.480
fG/E	1.467	1.454	1.484	1.023	1.469	1.454	1.464	0.501
fG/F	1.839	1.855	1.865	0.724	1.888	1.855	1.840	1.322
fG/H	2.188	2.199	2.223	0.809	2.254	2.199	2.206	1.342
fG/I	0.988	0.999	1.005	0.870	1.015	0.999	1.001	0.898

[3] Note: A: sibiricose A5; B: sibiricose A6; C: sibiricaxanthone B; D: glomeratose A; E: polygalaxanthone III; F: tenuifolisideB; G: 3,6′-disinapoyl sucrose (DISS); H: tenuifolisideA; I: tenuifolisideC.

Table 4. Different geographical locations of 23 *P. tenuifolia* samples in China.

Number	Province	Population	Collection Time	Number	Province	Population	Collection Time
S1	Shanxi	Xinjiang, Yuncheng	2013.9	S13	Shanxi	Aodi, Wenxi	2013.9
S2	Shanxi	Hongdong, Linfen	2013.7	S14	Shanxi	Zhangdian, Pinglu	2013.9
S3	Shanxi	Xiangfen, Linfen	2013.7	S15	Shanxi	Fenyang	2013.7
S4	Shanxi	Liyuan, Yuncheng	2013.9	S16	Shanxi	Niangzhangzhen, Xiaxian	2013.7
S5	Shanxi	Wan′an, Xinjiang	2013.9	S17	Shanxi	Beizhang, Taiyuan	2013.9
S6	Shanxi	Dongzhen, Wenxi	2013.9	S18	Shanxi	Xuedian, Wenxi	2013.9
S7	Shanxi	Lanxian, Lvliang	2013.9	S19	Shanxi	Houma	2013.9
S8	Shanxi	Hedi, Yangquan	2013.9	S20	Heibei	An′guo, Baoding	2013.7
S9	Shanxi	Daiyu, Jishan	2013.9	S21	Shaanxi	Suide, Yulin	2013.7

S10	Shanxi	Yonghe, Linfen	2013.7	S22	Shaanxi	Chengcheng, Weinan	2013.7
S11	Shanxi	Peishe, Wenxi	2013.9	S23	Shaanxi	Heyang, Weinan	2013.7
S12	Shanxi	Pinyao, Jinzhong	2013.7				
